# Coral Reef Health Indices *versus* the Biological, Ecological and Functional Diversity of Fish and Coral Assemblages in the Caribbean Sea

**DOI:** 10.1371/journal.pone.0161812

**Published:** 2016-08-31

**Authors:** Leopoldo Díaz-Pérez, Fabián Alejandro Rodríguez-Zaragoza, Marco Ortiz, Amílcar Leví Cupul-Magaña, Jose D. Carriquiry, Eduardo Ríos-Jara, Alma Paola Rodríguez-Troncoso, María del Carmen García-Rivas

**Affiliations:** 1 Laboratorio de Ecosistemas Marinos y Acuicultura, Departamento de Ecología, Centro Universitario de Ciencias Biológicas y Agropecuarias, Universidad de Guadalajara, Zapopan Jalisco, México; 2 Laboratorio de Modelamiento de Sistemas Ecológicos Complejos (LAMSEC), Instituto de Ciencias Naturales AvH, Facultad de Ciencias del Mar y Recursos Biológicos Investigaciones Oceanológicas, Facultad de Recursos del Mar, Universidad de Antofagasta, Antofagasta, Chile; 3 Departamento de Ciencias Biológicas, Centro de Investigaciones Costeras, Centro Universitario de la Costa, Universidad de Guadalajara, Puerto Vallarta, Jalisco, México; 4 Instituto de Investigaciones Oceanológicas, Universidad Autónoma de Baja California, Ensenada, Baja California, México; 5 Reserva de la Biosfera Banco Chinchorro- CONANP. Chetumal, Quintana Roo, México; Universita degli Studi di Genova, ITALY

## Abstract

This study evaluated the relationship between the indices known as the Reef Health Index (RHI) and two-dimensional Coral Health Index (2D-CHI) and different representative metrics of biological, ecological and functional diversity of fish and corals in 101 reef sites located across seven zones in the western Caribbean Sea. Species richness and average taxonomic distinctness were used to asses biological estimation; while ecological diversity was evaluated with the indices of Shannon diversity and Pielou´s evenness, as well as by taxonomic diversity and distinctness. Functional diversity considered the number of functional groups, the Shannon diversity and the functional Pielou´s evenness. According to the RHI, 57.15% of the zones were classified as presenting a "poor" health grade, while 42.85% were in "critical" grade. Based on the 2D-CHI, 28.5% of the zones were in "degraded" condition and 71.5% were "very degraded". Differences in fish and coral diversity among sites and zones were demonstrated using permutational ANOVAs. Differences between the two health indices (RHI and 2D-CHI) and some indices of biological, ecological and functional diversity of fish and corals were observed; however, only the RHI showed a correlation between the health grades and the species and functional group richness of fish at the scale of sites, and with the species and functional group richness and Shannon diversity of the fish assemblages at the scale of zones. None of the health indices were related to the metrics analyzed for the coral diversity. In general, our study suggests that the estimation of health indices should be complemented with classic community indices, or should at least include diversity indices of fish and corals, in order to improve the accuracy of the estimated health status of coral reefs in the western Caribbean Sea.

## Introduction

Coral reefs have suffered significant impacts over recent years as a result of the impact of human and natural disturbances on their biodiversity and ecosystem functioning [1–3]. These ecosystems are fragile and sensitive to disturbance and are at greater risk of degradation than other marine systems [4]. In recent decades, a rapid decline in coral coverage has been documented worldwide, with total losses of 19% of live coral and another 15% of the coral cover classified as seriously threatened [5]. This situation is the result of synergic factors such as overfishing, eutrophication of seawater by excessive input of nutrients, sedimentation, increased temperatures, ocean acidification, hurricanes and tropical storms, among others [6–11], and has generated an increase in coral bleaching events and mortalities, overgrowth of fleshy macroalgae, presence of diseases and a decline in physiological processes such as growth and calcification [12–14]. This has diminished the health or fitness of the coral reefs, which are now considered to be in crisis at a global level [15–16].

As a consequence, research exploring strategies of conservation, as well studies of the health of coral reefs, have increased considerably [17–19]. To date, reef health has been evaluated a range of indices and single indicators, including: total live coral cover and macroalgae cover [20], richness or number of species [21,22,23], Shannon diversity index (*H*’) [21,22,23], Pielou´s evenness index (*J’*) [21,22,23], Simpson´s dominance index (*D*) [24], coral mortality index [25], coral damage index (CDI) [26], reef conservation value [27], deterioration index (DI) [27], coral condition index (CCI) [27,28], reef condition index (RCI) [29]. Some of these indices, such as those of reef condition (RCI) and coral mortality, are now in disuse but the rest are still in application.

In the Caribbean Sea, the Healthy Reefs Initiative (HRI), formerly called the Healthy Mesoamerican Reef Ecosystem (HMRE), is an international and inter-institutional initiative focused on evaluation of reef health [18, 30] and collaborative evaluation of management efforts through Eco-Audits (See. www.healthyreefs.org); they developed a Reef Health Index (RHI), which combines four indicators for evaluation: live coral coverage, fleshy macroalgae coverage, biomass of herbivorous fish and biomass of commercial fish in order to convey one simplified measure of reef health to policy makers and the general public. These indicators are averaged in order to obtain values on a range from 1 to 5, where the value of 1 is characterized as “Critical”, value of 2 as “Poor”, value of 3 as “Fair”, value of 4 as “Good” and value of 5 as “Very Good” [31,32]. The recent “Mesoamerican Reef” (MAR) evaluations reported an increase in the percentage of reefs in critical condition, from 6% in 2008 to 30% in 2010. In a report of 2012, this latter value had decreased to 24% and an improvement in the health of the reefs was reported in 2015, since only 17% of the evaluated sites were still considered to be in critical condition at that time [33–34, 35].

The Coral Health Index (CHI) [36] is another indicator of coral reef health and has been used on the Line and Hawaiian Islands in the Central Pacific Ocean, on the East coast of Kenya and Tanzania in the Indian Ocean and in the Dutch Antilles in the Caribbean Sea [36]. This index uses three indicators: benthos (including the coverage values of both encrusting coralline algae and live coral), reef fish (total biomass of fish) and microbes (concentration of *Vibrio* spp.). These indicators are averaged in order to obtain five health grades in values ranging from zero to one, 0.0–0.20 (very degraded), 0.21–0.40 (degraded), 0.41–0.60 (Fair), 0.61–0.80 (healthy), 0.81–1.0 (very healthy). Due to the relative novelty of the bacterial component, there is little pertinent information available. However, Kaufman et al. (2011)[36] defined the two-dimensional Coral Health Index (2D-CHI), which was only estimated with data pertaining to the benthos and fish. This modification has been successfully used in many places in the Caribbean Sea and Pacific and Indian Oceans, indicating health differences between protected and non-protected areas and becoming an effective indictor with which to evaluate differences in temporal and spatial health [36].

The health status of coral reefs and other types of aquatic ecosystems has also been evaluated from a community ecology perspective that incorporates attributes such as species richness, relative abundance, species evenness, rare species and coral cover [37,38,39]. It considers changes in species diversity as an indicator of disturbance [40,41]. In this sense, biodiversity conservation favors the natural resilience of the coral reefs to recover from disturbance and maintain their ecosystem services [42]. Structural changes in the reef ecosystems affect their biodiversity (e.g. abundance of taxa) and, with this, their ecosystem processes [43]. These indices types consider the proportional abundances of taxa, based on different aspects of the diversity. They can be of statistical type, or involve information theory, dominance of species and taxonomic diversity [41]. In coral reefs, these metrics have been focused on the assemblages of reef fish and coral species [44–46]. This has revealed that decline in species diversity especially that of rare species is often associated with reduced coral cover and increased macroalgae [47–50].

An important advance is currently taking place in the assessment of coral reef health, supported mainly by community attributes such as biological, ecological and functional diversity indices. Nevertheless, no studies have been conducted to date that evaluate the relationship between the RHI and 2D-CHI indices and those of the biological, ecological and functional diversity of reef fish and coral assemblages. The RHI and 2D-CHI are commonly implemented by authorities, managers and institutions in order to evaluate the health and conservation of coral reefs in different parts of the world, but this remains to be assessed in many locations of the Mesoamerican Coral Reef. The objective of this study was therefore to determine the relationship between the RHI and 2D-CHI and indices of the biological, ecological and functional diversity of the assemblages of reef fish and hermatypic corals of different coral reefs of the western Caribbean [45, 46, 48, 49, 51].

## Materials and Methods

### Study area

**Fig 1:**
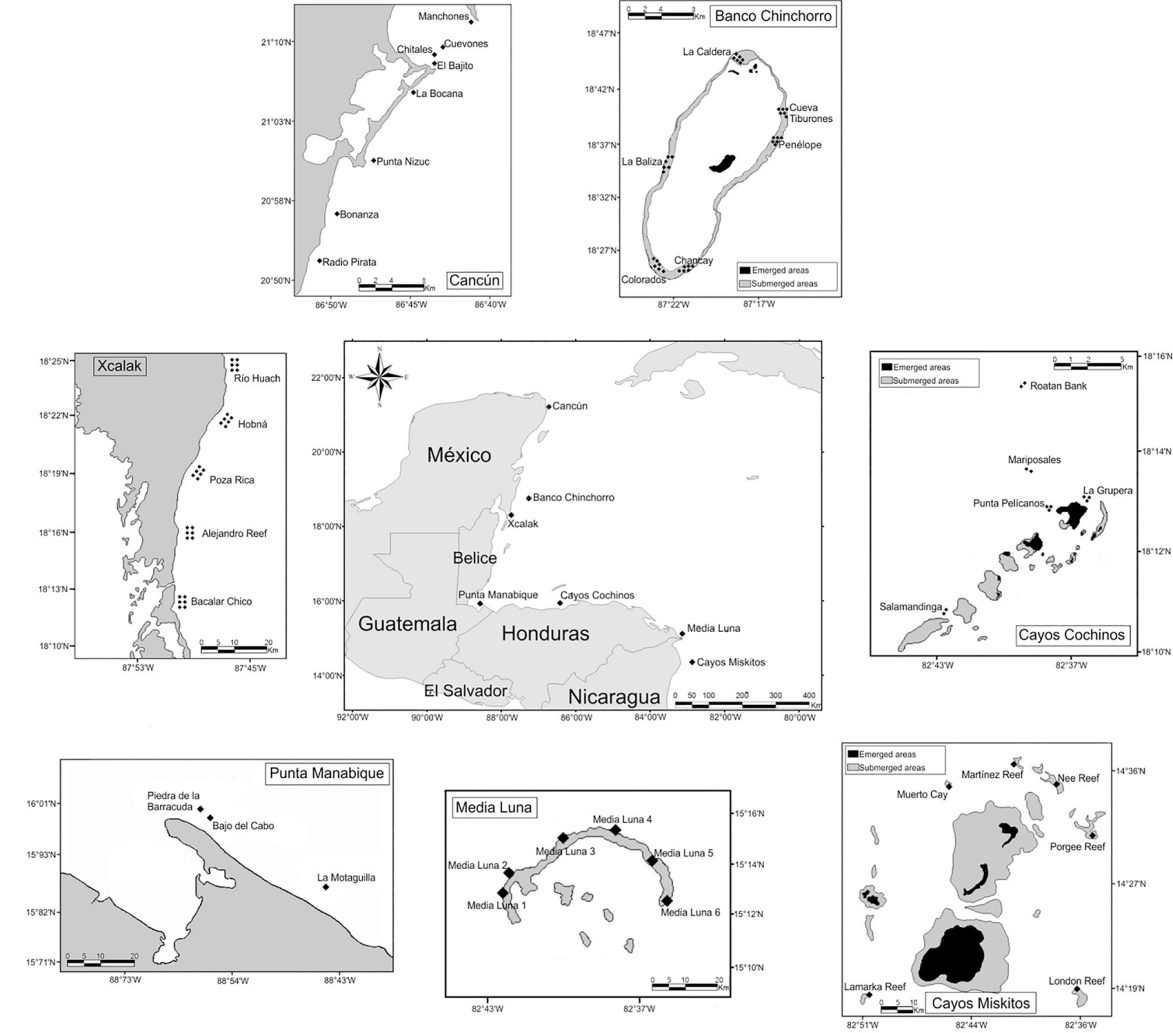
Study area with zones of coral reefs in the western Caribbean. Cancún, Banco Chinchorro, Xcalak, Punta Manabique, Cayos Cochinos Media Luna, Cayos Miskitos.

The study area covered the coasts of Mexico, Guatemala, Honduras and Nicaragua, where 101sampling sites in seven zones were evaluated. The first three of the aforementioned countries have territories on the Mesoamerican Reef and the fourth is part of the western Caribbean. The study zones were: Cancún, Banco Chinchorro and Xcalak (Mexico); Punta Manabique (Guatemala); Cayos Cochinos and Media Luna (Honduras); and Cayos Miskitos (Nicaragua) (Fig 1). The Mesoamerican Reef comprises the largest barrier reef in the western Caribbean. This system presents a large variety of reef structures, notable among which are four atolls located at a considerable distance from the coast. It extends over a distance of ~1000 km and includes the northern Yucatán Peninsula and the Belize, Guatemala and Honduras coastlines [49,52].

### Field and laboratory work

A hierarchical sampling design was conducted for each zone and its respective sampling sites. Sampling was conducted with the cooperation of different governmental institutions, non-governmental organizations (NGOs) and universities, including the Mexican National Commission for Protected Natural Areas (CONANP, by its Spanish acronym), The Nicaraguan Institute of Fishery (INPESCA, by its Spanish acronym), World Wildlife Fund-Central America (WWF-CA), Cayo Cochinos Foundation (Honduras), Mario Dary Foundation (Guatemala), University of Guadalajara (UDG, Mexico) and the Autonomous University of Baja California (UABC, Mexico).

A total of 507 transects were conducted between 2007 and 2011. At each transect, visual fish censuses (2 x 50 m) and characterization of the benthic structure using video transects (~0.8 x 50 m) with a underwater camera (JVC Everio-HD Hard Disk Camcorder and SONY Handycam HD AVCHD, resolution of 1920 x 1080i) were conducted in depths from 3 to ~30 m at all of the sampling sites (S1 Fig). The fish and benthic components were recorded simultaneously.

The fish censuses documented species richness, as well as size and abundance. Biomass was estimated per transect, site and zone using the exponential function *W = aL*^*b*^, where *W* is the biomass (g m^-2^), *a* and *b* correspond to the constants of the length-weight relationship obtained from the database FishBase® and *L* is the length value of the weighted average size [53]. Fish functional groups were determined based on their different trophic, morphological and functional characteristics (S1 Table) [54].

For each video transect, 40 quadrants (~0.8 x ~0.6 m) and 13 fixed points were examined using conventional video-processing software (CyberLink PowerDVD 13) in order to estimate the richness, cover and functional groups of hermatypic corals, as well as the coverage of fleshy and encrusting coralline algae [48,55]. Coral colonies were classified using the criteria of Arias-González et al. (2008) [45], based on the morphology of the colony (i.e. semispherical, boulder, encrusting, foliose, fleshy, branching, columnar, finger, plate and brain corals).

### Data analysis

The RHI, developed by Healthy Reefs for Healthy People [31–34], was estimated using four indicators: i) live coral cover; ii) fleshy macroalgae cover; iii) biomass of herbivorous fish (g 100 m^-2^), of the families Scaridae and Acanthuridae; and iv) biomass of commercial fish (g 100 m^-2^), using only the families Lutjanidae and Serranidae. These families of fish were used because of their important functional role within coral reefs; the scarids and acanthurids are considered the most important grazers within the reefs, since they reduce the overgrowth of fleshy macroalgae. Snappers (Lutjanidae) and serranids are of important commercial value, but also play an important trophic role as carnivores that exert a strong predatory influence in the coral reefs. The mean value of each indicator was converted to an ordinal scale, with values of 1 (“critical”) to 5 (“very good”), producing five grades of health (S2 Table) [31–33]. The 2D-CHI index developed by Kaufman et al. (2011) was estimated based on the combination of two diagnostic parameters: benthos and fish [36]. Due to the lack of pertinent data, a bacterial diagnostic parameter was not used in the present study [36]. The diagnostic parameters were scaled from a value of zero (“very degraded”) to one (“very healthy”), producing five grades of health (S3 Table). Estimation of the benthos was obtained using the mean live coral and encrusting coralline algae values and dividing by 100. Estimation of the fish considered the total biomass (g m^-2^) as a fraction of 500 g m^-2^, with subsequent averaging of the values of each parameter in order to obtain the final 2D-CHI value [36].

The biological, ecological and functional diversity of the fish and coral components was estimated (separately) for each biological group at transect scale, and mean values were then calculated at sampling site and reef zone scale. The analysis of biological diversity considered species richness (S) and average taxonomic distinctness (∆^+^) [56–58]. The trajectory lengths were weighted: ω = 1, species of the same genus; ω = 2, species of the same family but of a different genus; ω = 3, species of the same order but of a different family and genus; ω = 4, species of the same class but of a different order, family and genus [58]. Ecological diversity was evaluated using Shannon diversity (*H*’, nats), Pielou´s evenness (*J’*), taxonomic diversity (∆) and taxonomic distinctness (∆*) [58–59]. Functional diversity considering functional richness (= number of functional groups) and functional heterogeneity was estimated with Shannon diversity and functional Pielou´s evenness [60].

Different statistical models were used to evaluate the relationship between the reef health indices (RHI and 2D-CHI) and the representative metrics of biodiversity. Multi-scale analyses were applied considering the biological, ecological and functional spatial variation among sites, reef zones and degrees of health of the RHI or 2D-CHI indices, through permutational analysis of variance (ANOVA) based on matrices of Euclidean distances [61]. This non-restricted method was utilized since the data did not fulfill the criteria for parametric statistics [61–62]. The factors considered in the models were: sites (random effect, 101 sites), zones (random effect, seven zones) and grades of health in the RHI (fixed effect, three or four grades) and 2D-CHI (fixed effect, two or three grades). All of the models analyzed were thus of mixed effects in type.

Model 1 was a completely nested two-way ANOVA design: Y = zones_i_ + sites_j_(zones_i_) + ε_ij_, where Y is the dependent variable. This design analyzed the spatial variation of the representative metrics of fish and coral diversity (Y) among sampling sites and reef zones. Similarly, model 2 considered a nested two-way ANOVA, Y = health grade_i_ + site_j_(health grade_i_) + ε_ij_, which explained the variation in the biological, ecological and functional diversity among different health grade (RHI and 2D-CHI, separately) at the scale of sites. Model 3 corresponded to a nested three-way ANOVA, Y = health grade_i_ + zones_j_(health grade_i_) + sites_k_(zones_i_(health grade_i_)) + ε_ijk_, which explained the variation of the metrics of diversity used among the different health grade estimated by RHI and 2D-CHI separately at the scales of site and zone.

The relationship between RHI and 2D-CHI, with respect to the metrics of biological, ecological and functional diversity, was evaluated through linear and non-linear regressions using an exponential raised to maximum with 2 parameters function (*Y* = *a*(1 − *e*^−*bx*^)), where the RHI and 2D-CHI were the independent variables and the different diversity types were the dependent variables. In addition, Pearson correlations were used among all of these variables at the scales of site and zone. All of the analyses of biological, ecological and functional diversity, as well as the statistical models, were performed using the software Primer 6 + Permanova. The linear and non-linear regressions were performed using SigmaPlot 11®.

## Results

### Reef health at the scales of sites and zones

#### Reef health at the scale of sites

**Fig 2:**
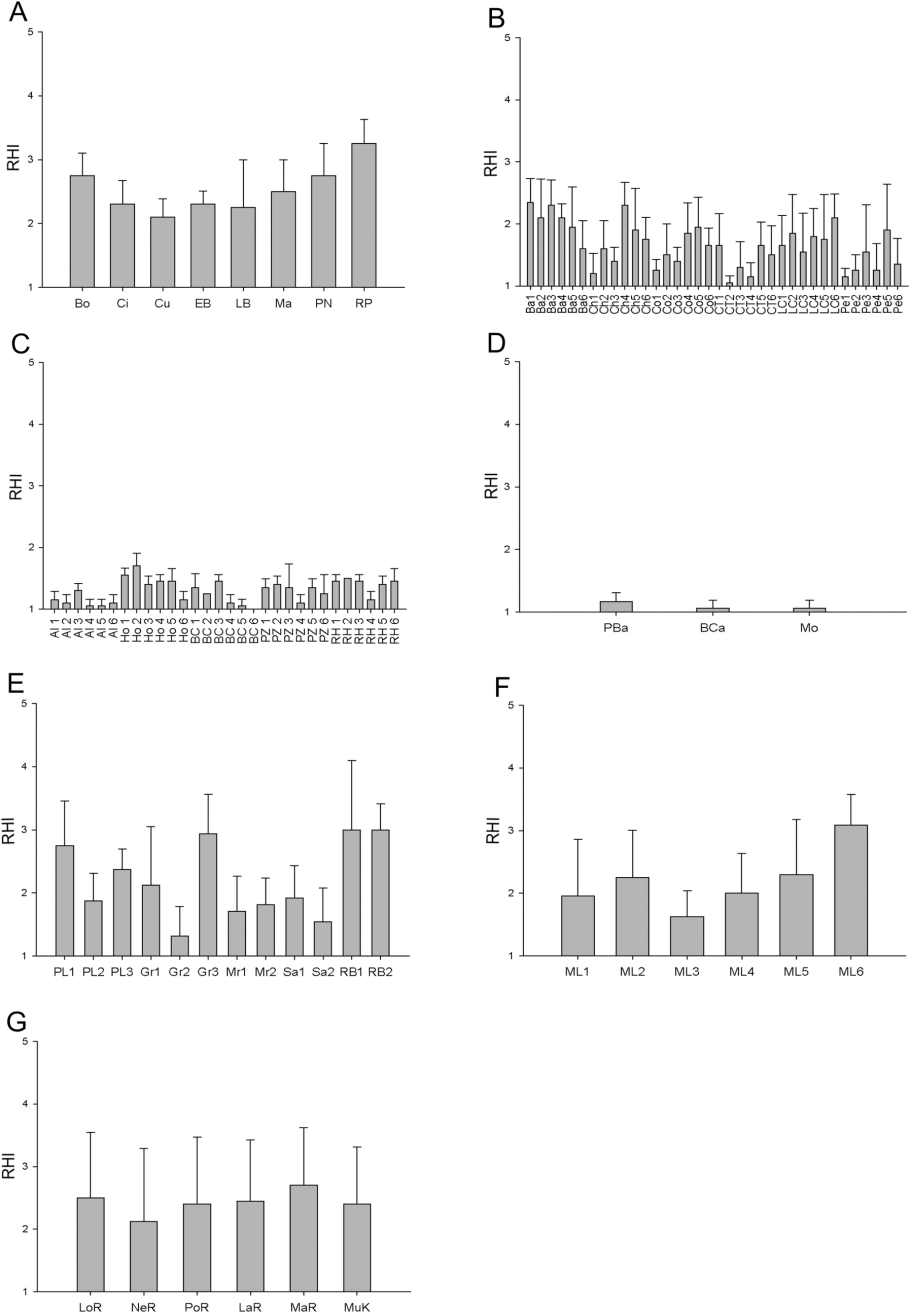
Coral reef health in sampling sites evaluated with RHI. **a) Cancun:** Bonanza (Bo), Chitales (Ci), Cuevones (Cu), El Bajito (EB), La Bocana (LB), Manchones (Ma), Punta Nizuc (PN), Radio Pirata (RP); **b) Banco Chinchorro:** Six sites for Baliza (Ba1, Ba2, Ba3, Ba4, Ba5, Ba6), six sites for Chancay (Ch1, Ch2, Ch3, Ch4, Ch5, Ch6), six sites for Colorados (Co1, Co2, Co3, Co4, Co5, Co6), six sites for Cueva Tiburones (CT1, CT2, CT3, CT4, CT5, CT6), six sites for La Caldera (LC1, LC2, LC3, LC4, LC5, LC6), and six sites for Penelope (Pe1, Pe2, Pe3, Pe4, Pe5, Pe6); **c) Xcalak:** six sites for Alejandro Reefs (Al1, Al2, Al3, Al4, Al5, Al6), six sites for Hobná (Ho1, Ho2, Ho3, Ho4, Ho5, Ho6), six sites for Bacalar Chico (Bac1, Bac2, Bac3, Bac4, Bac5, Bac6), six sites for Poza Rica (PZ1, PZ2, PZ3, PZ4, PZ5, PZ6), and six sites for Rio Huach (RH1, RH2, RH3, RH4, RH5, RH6); **d) Punta Manabique:** Piedra de la barracuda (PBA), Bajo del Cabo (BCA), Motaguilla (Mo); **e) Cayos Cochinos:** three sites for Pelicanos (PL1, PL2, PL3), three sites for Grupera (Gr1, Gr2, Gr3), two sites for Mariposales (Mr1, Mr2), two sites for Salamandinga (Sa1, Sa2), and two sites for Roatan Bank (RB1, RB2); **f) Media Luna:** six sites for Media Luna (ML1, ML2, ML3, ML4, ML5, ML6) and **g) Cayos Miskitos:** London Reef (LOR), Nee Reef (NER), Porgee Reef (POR), Lamarka Reef (LAR), Martinez Reef (MAR), Cayo Muerto (MUK). Error bars correspond to standard deviation value.

**Fig 3:**
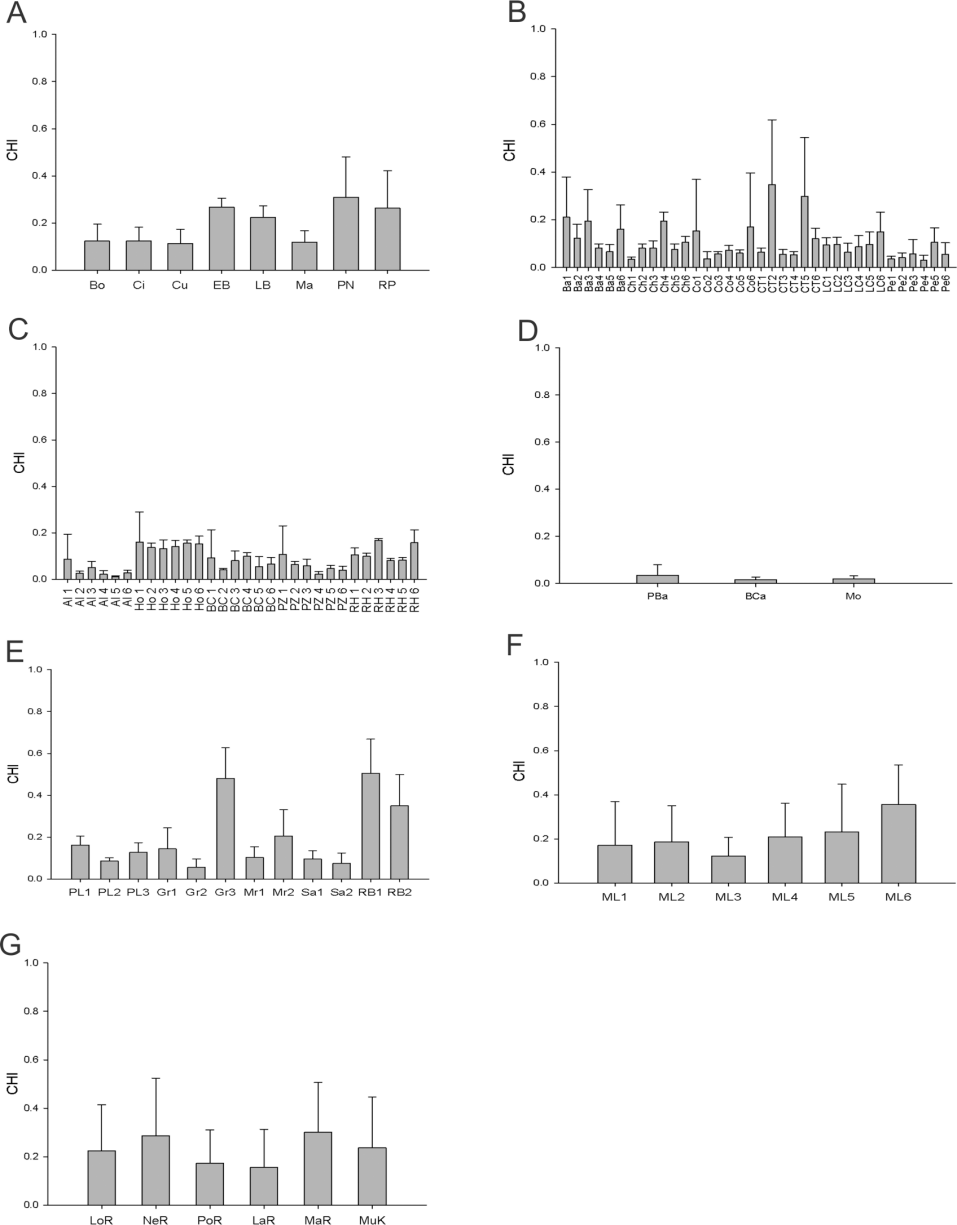
Coral reef health in sampling sites evaluated with CHI. **a) Cancun:** Bonanza (Bo), Chitales (Ci), Cuevones (Cu), El Bajito (EB), La Bocana (LB), Manchones (Ma), Punta Nizuc (PN), Radio Pirata (RP); **b) Banco Chinchorro:** Six sites for Baliza (Ba1, Ba2, Ba3, Ba4, Ba5, Ba6), six sites for Chancay (Ch1, Ch2, Ch3, Ch4, Ch5, Ch6), six sites for Colorados (Co1, Co2, Co3, Co4, Co5, Co6), six sites for Cueva Tiburones (CT1, CT2, CT3, CT4, CT5, CT6), six sites for La Caldera (LC1, LC2, LC3, LC4, LC5, LC6), and six sites for Penelope (Pe1, Pe2, Pe3, Pe4, Pe5, Pe6); **c) Xcalak:** six sites for Alejandro Reefs (Al1, Al2, Al3, Al4, Al5, Al6), six sites for Hobná (Ho1, Ho2, Ho3, Ho4, Ho5, Ho6), six sites for Bacalar Chico (Bac1, Bac2, Bac3, Bac4, Bac5, Bac6), six sites for Poza Rica (PZ1, PZ2, PZ3, PZ4, PZ5, PZ6), and six sites for Rio Huach (RH1, RH2, RH3, RH4, RH5, RH6); **d) Punta Manabique:** Piedra de la barracuda (PBA), Bajo del Cabo (BCA), Motaguilla (Mo); **e) Cayos Cochinos:** three sites for Pelicanos (PL1, PL2, PL3), three sites for Grupera (Gr1, Gr2, Gr3), two sites for Mariposales (Mr1, Mr2), two sites for Salamandinga (Sa1, Sa2), and two sites for Roatan Bank (RB1, RB2); **f) Media Luna:** six sites for Media Luna (ML1, ML2, ML3, ML4, ML5, ML6) and **g) Cayos Miskitos:** London Reef (LOR), Nee Reef (NER), Porgee Reef (POR), Lamarka Reef (LAR), Martinez Reef (MAR), Cayo Muerto (MUK). Error bars correspond to standard deviation.

The RHI index showed that the health condition of the coral reefs of Cancún Mexico was fair in Radio Pirata and poor in Bonanza, Chitales, Cuevones, El Bajito, La Bocana, Punta Nizuc and Manchones (Fig 2A). The 2D-CHI indicated that the health grade of El Bajito, La Bocana, Punta Nizuc and Radio Pirata was degraded, while the remaining sites were very degraded (Fig 3A). According to the RHI, the sites Chancay, Colorados, Cueva Tiburones, La Caldera and Penélope in the zone of Banco Chinchorro were in critical condition and only La Baliza presented a poor condition of health (Fig 2B). However, the 2D-CHI analysis indicated that all of these sites presented a very degraded condition of health (Fig 3B). According to the RHI and 2D-CHI, the conditions of the sites of the zone of Xcalak were critical and very degraded, respectively (Figs 2C and 3C).

In the zone of Punta Manabique, Guatemala, all of the sites were in a critical condition according to the RHI (Fig 2D), while the 2D-CHI indicated that these sites were in very degraded condition (Fig 3D). In the zone of Cayos Cochinos (Honduras), the RHI analysis showed that Roatan Bank presented a fair health grade, while the sites Pelicanos and La Grupera were in poor condition and Mariposales and Salamandinga were in critical condition (Fig 2E). According to the 2D-CHI, Roatan Bank was degraded, while the other sites were very degraded (Fig 3E). In the reefs of Media Luna in Honduras, the RHI results indicated three sites with a critical health grade (ML1, ML3 and ML4), with a further two classified as poor (ML2 and ML5) and one with fair condition (ML6)(Fig 2F). Evaluation with the 2D-CHI showed that three of the sites were in a very degraded health grade (ML1, ML2 and ML3), while the other three sites were degraded (Fig 3F). Finally, the sites of Cayos Miskitos, Nicaragua were in poor condition according to the RHI (Fig 2G), however, the results obtained from the 2D-CHI indicated that Porgee Reef and Lamarka Reef were in a very degraded health grade while the rest of the sites were degraded (Fig 3G).

#### Reef health at the scale of zones

**Fig 4:**
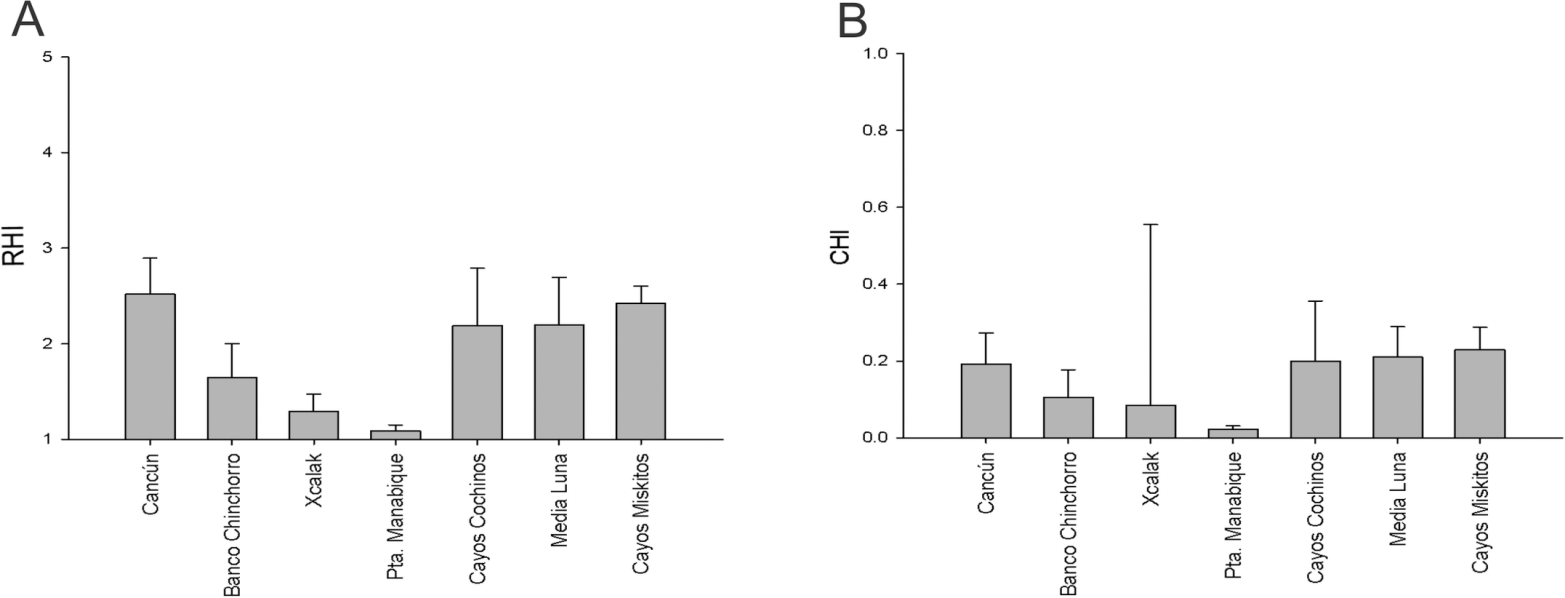
Grade of reef health estimated with the indices RHI and 2D-CHI in each study zone. **a)** RHI health grade per zone; **b)** CHI health grade per zone.

The RHI showed that Media Luna, Cayos Miskitos, Cayos Cochinos and Cancun coral reef presented a poor health grade, with Cancun and Cayos Miskitos presenting the best health of all of these reefs. The worst conditions were observed at Banco Chinchorro, Xcalak and Punta Manabique presented a critical health grade (Fig 4A). The 2D-CHI indicated a degraded condition in Media Luna, and Cayos Miskitos, where although Media Luna once again presented the best health grade of this group. In contrast, Xcalak, Banco Chinchorro, Cayos Cochinos, Cancun and Punta Manabique presented an outstanding degraded health grade, with Punta Manabique presenting the highest degradation (Fig 4B). In general, the RHI found that 57.15% of the reef zones presented a poor health and 42.85% were in critical condition, but the 2D-CHI indicated that 28.5% and 71.5% of the zones presented degraded and very degraded conditions, respectively.

### Indices of health *vs*. biological, ecological and functional diversity

The results of Model 1 showed that the majority of the indices of the biological, ecological and functional diversity of fish and coral varied significantly among zones and among the sites within each zone (Table 1). Nevertheless, no differences were observed between zones and sites with respect to the taxonomic diversity (Δ) estimated for fish and corals, nor in the Pielou´s evenness estimated for corals among zones (Table 1). However, the indices of functional diversity did show significant differences at the scales of zone and site (Table 1).

**Table 1:** Two-way nested permutational ANOVAs (Model 1) outcomes contrasting the biological, ecological and functional diversity of fish and hermatypic corals among zones and sites nested in zones.

**Variation source**	**Biological diversity**				**Ecological diversity**							
	***S***		***Δ***^***+***^		***H’***		***J’***		***Δ***		Δ*	
	Pseudo-F	*P*	Pseudo-F	*P*	Pseudo-F	*P*	Pseudo-F	*P*	Pseudo-F	*P*	Pseudo-F	*P*
***Reef Fish***												
Zones	24.427	**0.0001**	14.705	**0.0001**	6.3219	**0.0001**	5.6263	**0.0001**	0.3506	0.5437	17.29	**0.0001**
Sites (Zones)	3.849	**0.0001**	2.7999	**0.0001**	2.2506	**0.0001**	1.5905	**0.0014**	1.0697	0.456	2.1852	**0.0001**
***Hermatypic coral***												
Zones	17.774	**0.0001**	6.2241	**0.0002**	3.0795	**0.0087**	0.6169	0.7167	2.0519	0.0747	4.2537	**0.0013**
Sites (Zones)	6.008	**0.0001**	4.1346	**0.0001**	10.422	**0.0001**	9.0226	**0.0001**	0.9355	0.622	5.6755	**0.0001**
	**Functional diversity**											
	***S***		***H’***		***J’***							
	Pseudo-F	*P*	Pseudo-F	*P*	Pseudo-F	*P*						
***Reef Fish***												
Zones	16.481	**0.0001**	4.5633	**0.0006**	5.9222	**0.0001**						
Sites (Zone)	3.8058	**0.0001**	2.2081	**0.0001**	1.7649	**0.0001**						
***Hermatypic coral***												
Zones	19.246	**0.0001**	3.3803	**0.0063**	3.3919	**0.0059**						
Sites (Zones)	4.2912	**0.0001**	7.5326	**0.0001**	6.7442	**0.0001**						

Numbers in bold text denote significant differences (*P* < 0.05). Codes: (*S*) species richness, (*∆*^*+*^) average taxonomic distinctness, (*H’*) Shannon diversity, (*J’*) Pielou’s evenness, (∆) taxonomic diversity, and (∆*) taxonomic distinctness.

Model 2 showed that the majority of the indices representative of the fish and coral diversity (separately) did not present significant differences among the categories of the RHI and 2D-CHI, except for the average richness of species and functional groups of fish. Moreover, species evenness differed significantly among the health grades of both indices. Functional Pielou´s evenness also differed among the health grades produced by the 2D-CHI (Table 2). The functional Shannon diversity of the hermatypic corals varied significantly among the health grades of the RHI and 2D-CHI, while functional Pielou´s evenness only differed with the RHI (Table 2). In contrast, the analyses conducted among sites per of health grade (RHI-2D-CHI) showed that the majority of indices representative of the diversity of fish and corals differed significantly among sites (Table 2).

**Table 2:** Two-way nested and permutational ANOVAs (Model 2) outcomes contrasting the biological, ecological and functional diversity of fish and corals among different health grades of the RHI and 2D-CHI indices and among sites nested in the health indices.

**Variation source**	**Biological diversity**				**Ecological diversity**							
	***S***		***Δ***^***+***^		***H’***		***J’***		***Δ***		Δ*	
	Pseudo-F	*P*	Pseudo-F	*P*	Pseudo-F	*P*	Pseudo-F	*P*	Pseudo-F	*P*	Pseudo-F	*P*
***Reef Fish***												
RHI	20.736	**0.0001**	1.7173	0.1796	2.4398	0.0954	5.1449	**0.0067**	0.2210	0.9505	0.7940	0.4586
Sites (RHI)	7.2413	**0.0001**	5.2003	**0.0001**	2.9731	**0.0001**	2.0722	**0.0001**	1.0035	0.478	4.4203	**0.0001**
2D-CHI	3.8577	**0.0226**	0.7051	0.515	0.2383	0.786	3.6921	**0.0304**	0.0721	0.8413	0.9930	0.3794
Sites (2D -CHI)	9.4516	**0.0001**	5.3167	**0.0001**	3.0397	**0.0001**	1.9822	**0.0001**	1.0193	0.6757	4.4532	**0.0001**
***Hermatypic coral***												
RHI	1.3886	0.2525	2.8378	0.0624	0.9322	0.3989	1.824	0.1757	0.6338	0.9407	1.8693	0.1536
Sites (RHI)	11.494	**0.0001**	5.1382	**0.0001**	11.658	**0.0001**	8.6926	**0.0001**	1.0179	0.365	6.6202	**0.0001**
2D -CHI	0.0206	0.9717	2.4528	0.0994	1.6182	0.2034	1.1511	0.3119	0.0206	0.9717	2.4528	0.0994
Sites (2D -CHI)	1.0174	0.3492	6.443	**0.0001**	11.272	**0.0001**	11.592	**0.0001**	1.0174	0.3492	6.443	**0.0001**
	**Functional diversity**											
	***S***		***H’***		***J’***							
	Pseudo-F	*P*	Pseudo-F	*P*	Pseudo-F	*P*						
***Reef Fish***												
RHI	24.353	**0.0001**	2.6321	0.0784	1.9346	0.1472						
Sites (RHI)	5.2607	**0.0001**	2.621	**0.0001**	2.3376	**0.0001**						
2D -CHI	4.0731	**0.02**	0.4497	0.6302	4.7048	**0.0104**						
Sites (2D -CHI)	7.2405	**0.0001**	2.7497	**0.0001**	2.2092	**0.0001**						
***Hermatypic coral***												
RHI	0.0082	0.9918	3.2041	**0.0481**	3.6034	**0.03**						
Sites (RHI)	8.9328	**0.0001**	8.2211	**0.0001**	7.2787	**0.0001**						
2D -CHI	1.7352	0.1844	4.2175	**0.0191**	2.9747	0.059						
Sites (2D -CHI)	8.5835	**0.0001**	7.9496	**0.0001**	7.2869	**0.0001**						

Numbers in bold text denote significant differences (*P* < 0.05). Codes: (*S*) species richness, (*∆*^*+*^) average taxonomic distinctness, (*H’*) Shannon diversity, (*J’*) Pielou´s evenness, (∆) taxonomic diversity, and (∆*) taxonomic distinctness.

No significant differences were observed with respect to the taxonomic diversity of fish and corals in both health indices and no differences were observed in the average coral richness (Table 2).

Model 3 showed that the majority of the indices of the biological, ecological and functional diversity of fish and corals (separately) did not differ significantly among the categories of the RHI and 2D-CHI (Tables 3 and 4), except for the taxonomic distinctness of fish among health grades of the RHI and functional Shannon diversity of corals in the 2D-CHI. The analyses among zones per health grade of the RHI or 2D-CHI also revealed significant differences in most of the diversity indices for fish (Table 3) and corals (Table 4).

**Table 3:** Three-way fully nested and permutational ANOVAs outcomes contrasting biological, ecological and functional diversity of reef fish among different health grades of the RHI and 2D-CHI indices, among zones nested in health grades and among sites nested in zones and health grades.

**Variation source**	**Biological diversity**				**Ecological diversity**							
	***S***		***Δ***^***+***^		***H’***		***J’***		***Δ***		Δ*	
	Pseudo-F	*P*	Pseudo-F	*P*	Pseudo-F	*P*	Pseudo-F	*P*	Pseudo-F	*P*	Pseudo-F	*P*
RHI	2.4588	0.1701	4.9596	0.0537	1.2509	0.4322	0.2352	0.9897	5.8538	0.5281	9.5052	**0.0166**
Zone(RHI)	6.5162	**0.0005**	3.3511	**0.0146**	4.172	**0.0033**	4.0963	**0.0035**	0.1210	0.4265	1.9205	0.1173
Site(Zone(RHI))	3.849	**0.0001**	2.7999	**0.0001**	2.3315	**0.0001**	1.5905	**0.0018**	1.0697	0.4655	2.1852	**0.0001**
2D-CHI	1.8979	0.1825	1.2393	0.2892	2.2617	0.1481	0.0547	0.8233	0.3387	0.5222	0.7912	0.3862
Zone(2D -CHI)	24.632	**0.0001**	13.588	**0.0001**	6.6276	**0.0001**	6.0869	**0.0001**	0.3387	0.5622	16.789	**0.0001**
Site(Zone(2D -CHI))	3.849	**0.0001**	2.7999	**0.0001**	2.3315	**0.0001**	1.5905	**0.0016**	1.0697	0.4528	2.1852	**0.0002**
	**Functional diversity**											
	***S***		***H’***		***J’***							
	Pseudo-F	*P*	Pseudo-F	*P*	Pseudo-F	*P*						
RHI	3.3715	0.1095	1.35	0.4084	0.1802	0.9973						
Zone(RHI)	3.4718	**0.0083**	4.2062	**0.0029**	7.2528	**0.0001**						
Site(Zone(RHI))	3.8058	**0.0001**	2.2081	**0.0001**	1.7649	**0.0002**						
2D -CHI	2.6191	0.1192	4.1569	**0.0425**	0.6198	0.448						
Zone(2D -CHI)	15.348	**0.0001**	4.8306	**0.0012**	6.9128	**0.0001**						
Site(Zone(2D -CHI))	3.8058	**0.0001**	2.2081	**0.0001**	1.7649	**0.0001**						

Numbers in bold text denote significant differences (*P* < 0.05). Codes: (*S*) species richness, (*∆*^*+*^) average taxonomic distinctness, (*H’*) Shannon diversity, (*J’*) Pielou´s evenness, (∆) taxonomic diversity, and (∆*) taxonomic distinctness.

**Table 4:** Three-way fully nested and permutational ANOVAs outcomes contrasting the biological, ecological and functional diversity of hermatypic corals among different health grades of RHI and 2D-CHI indices, among zones nested in health grades and among sites nested in zones and health grades.

**Variation source**	**Biological diversity**				**Ecological diversity**							
	***S***		***Δ***^***+***^		***H’***		***J’***		***Δ***		Δ*	
	Pseudo-F	*P*	Pseudo-F	*P*	Pseudo-F	*P*	Pseudo-F	*P*	Pseudo-F	*P*	Pseudo-F	*P*
RHI	0.045	1.0	0.6151	0.7764	0.2408	0.9854	0.7499	0.6891	0.5953	0.7963	0.5843	0.7997
Zone(RHI)	17.707	**0.0001**	7.2101	**0.002**	4.2793	**0.0038**	0.9294	0.4416	2.2521	0.0825	5.518	**0.0004**
Site(Zone(RHI))	6.0081	**0.0001**	4.1346	**0.0001**	10.422	**0.0001**	9.0226	**0.0001**	0.9355	0.617	5.6755	**0.0001**
2D-CHI	0.0331	0.8583	2.9872	0.0953	0.2504	0.629	0.1923	0.6693	1.7981	0.1942	3.1155	0.0904
Zone(2D -CHI)	20.399	**0.0001**	6.9844	**0.0001**	3.6695	**0.0034**	0.7429	0.5863	2.3387	0.0573	4.748	**0.0012**
Site(Zone(2D -CHI))	6.0081	**0.0001**	4.1346	**0.0001**	10.422	**0.0001**	9.0226	**0.0001**	0.9355	0.6284	5.6755	**0.0001**
	**Functional diversity**											
	***S***		***H’***		***J’***							
	Pseudo-F	*P*	Pseudo-F	*P*	Pseudo-F	*P*						
RHI	0.2133	0.99	0.2461	0.9823	0.2828	0.9726						
Zone(RHI)	15.753	**0.0001**	4.5529	**0.0021**	2.9725	**0.0259**						
Site(Zone(RHI))	4.2912	**0.0001**	7.5326	**0.0001**	6.7442	**0.0001**						
2D -CHI	0.4848	0.5039	0.7428	0.4066	0.0119	0.9161						
Zone(2D -CHI)	23.113	**0.0001**	3.6276	**0.0061**	3.7643	**0.0027**						
Site(Zone(2D -CHI))	4.2912	**0.0001**	7.5326	**0.0001**	6.7442	**0.0001**						

Numbers in bold text denote significant differences (*P* < 0.05). Codes: (*S*) species richness, (*∆*^*+*^) average taxonomic distinctness, (*H’*) Shannon diversity, (*J’*) Pielou´s evenness, (∆) taxonomic diversity, and (∆*) taxonomic distinctness.

No differences were found in the taxonomic diversity of fish in both health indices, or in their taxonomic distinctness with the RHI. Regarding the corals, Pielou´s evenness and taxonomic diversity did not present differences in either of the two health indices (Table 4). Finally, at the scale of sites nested per zone, most of the analysis showed significant differences among the indices of biological, ecological and functional diversity; however, no difference was detected in the taxonomic diversity of the fish (Table 3) or corals (Table 4).

**Fig 5:**
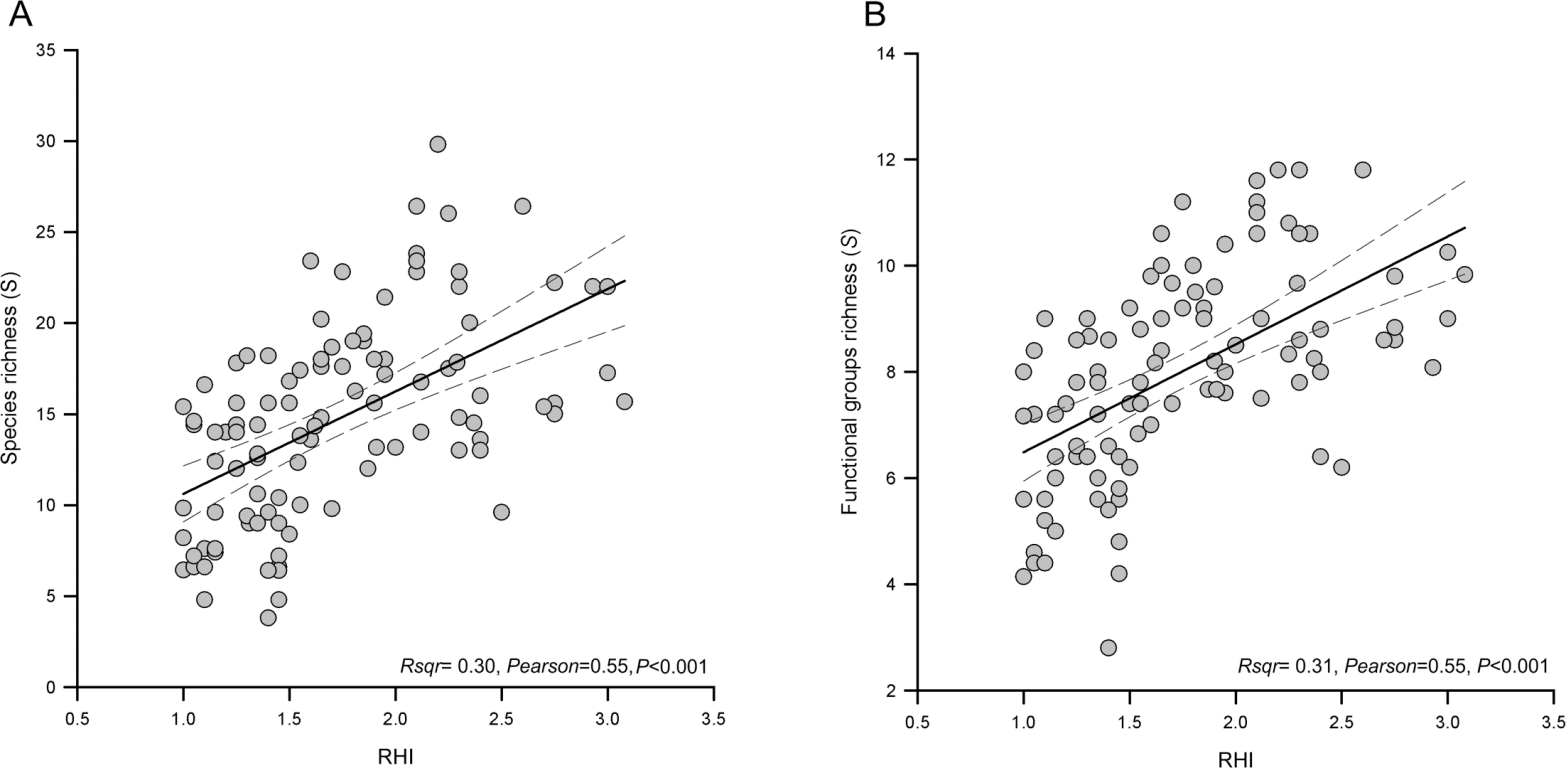
Linear regression and Pearson correlation. a) species richness of fish, b) richness of functional groups of fish in relation to the grades of coral health, according to the RHI at the scale of sites. Dotted lines indicate the 95% confidence interval.

**Fig 6:**
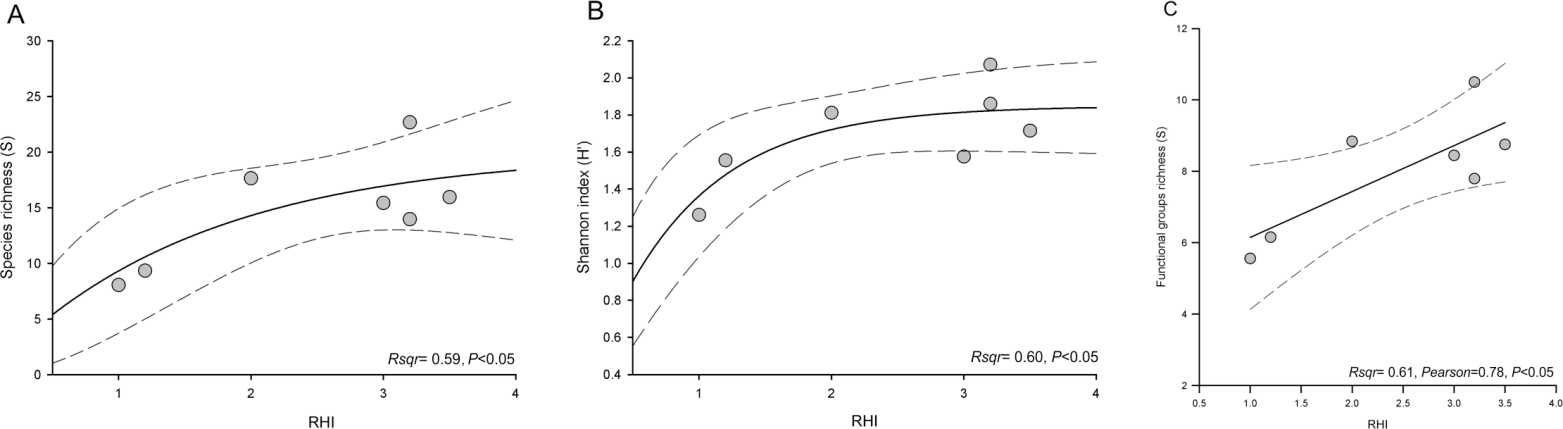
Relationship between RHI and the biological, ecological and functional indices of fish diversity. a) species richness, b) Shannon diversity, c) richness of functional groups of fish according to the grades of RHI at the scales of zones. Fig 6A and 6B present nonlinear regressions using an exponential raised to maximum with 2 parameters function (equation Fig 6A: f = 19.97(1-exp(-0.62x)), Fig 6B: f = 19.97(1-exp(-0.62x))) to get the best fit of the data. c) Linear regression and Pearson correlation. Dotted lines indicate the 95% confidence interval.

The results of the linear and non-linear regressions showed that the health grades of the 2D-CHI were not related to the diversity indices of fish and corals (separately) at either zone or site scale. In contrast, the health grades of the RHI presented a weak but significant relationship with species richness and the functional groups of fish only at the scale of site (Fig 5) and zones (Fig 6A and 6B), as well as with the Shannon diversity among zones (Fig 6C).

## Discussion

### Reef health indices

This study revealed very similar health profiles at site and zone scales using both health indices. However, the RHI presented higher reef health values in all zones and sites compared to 2D-CHI. The RHI analysis suggests that 57.15% of the zones presented poor health, while 42.85% were in critical condition. In contrast, the 2D-CHI estimates showed higher degradation in all of the studied sites, since 28.5% were considered to be degraded and 71.5% to be very degraded health. This discrepancy between the two indices is the result of differences in the variables considered for estimating reef health. The 2D-CHI considers the total fish biomass, as well as the coverage of encrusting coralline algae, while the RHI only considers the biomass of the herbivorous fish and species of commercial importance, limited to specific families, without considering encrusting coralline algae. It should be noted, however, that both reef health indices consider total live coral cover [32,33,36]. In general, both indices indicated that Media Luna, Cayos Miskitos, and Cancun were the zones that presented the best health condition (i.e. poor status) and the reports from Healthy Reefs for Healthy People during the years 2008, 2010 and 2012, in the reefs of Cancun, present similar results where the RHI was the index utilized [31–33]. It is important to highlight the absence of previous health reports using the indices RHI and 2D-CHI for the sites of Media Luna and Cayos Miskitos, since these sites are not considered part of the Mesoamerican Reef [34,36]. The zone of Banco Chinchorro presented a “critical” health status under the RHI and “very degraded” with the 2D-CHI, this was not similar to that reported for these sites in the years 2010 and 2012 [32–33]. However, both health indices indicate that both Xcalak and Punta Manabique present the worst health conditions, with grades of “critical” and “very degraded” under the RHI and 2D-CHI, respectively. This coincides with the RHI-based report of 2008 in Punta Manabique [31] and that of 2012 in Xcalak [33].

### Biological, ecological and functional diversity *vs*. RHI and 2D-CHI

This study showed a positive correlation between the species richness, functional group richness and Shannon diversity of fish at the scales of site and zone and the different health grades of the RHI. This is likely to be a consequence of the cause/effect relationship between the live coral coverage and fish species richness [42,45,46,49]. Thus far, it has been established that changes in the coverage of live coral promote significant changes in the richness and abundance of fish associated to these corals [60–61]. It is estimated that around 60% of the species of reef fish inhabit coral reef sites, since they depend on the resources provided by the corals, such as food, refuge and reproduction/recruitment sites. The presence of coral is therefore a factor that strongly influences the composition of fish species [60–61]. Specialized fish species, such as the corallivores, depend on specific coral species for food and habitat [62]. However other organisms associated with the corals also find food, refuge and zones for reproduction [63,64]. Our analyses at the scales of site and zone do not reveal any correlation between the two health indices and the metrics of biological, ecological and functional diversity of the hermatypic corals. This can be explained by the fact that neither indices considers the species richness or functional groups of the corals, although the RHI exclusively considers these attributes in the reef fish [31,33,36]. Consideration must also be given to disturbance theory [40], which explains that sites with minor disturbance will become dominated by the most abundant coral genera (e.g. *Orbicella*, *Siderastrea*, *Agaricia*, *Pseudodiploria*), that a high live coral coverage will result in high reef health values in both RHI and 2D-CHI indices and, in contrast, that a low coral diversity or richness will be associated with a disturbed site with lower live coral cover values.

While the RHI and 2D-CHI were developed in order to estimate the current status of the reef ecosystems and as a tool for management or conservation strategies [31–34,36], they exclude relevant ecological information that is necessary for the estimation of the coral reef status in greater detail. Nevertheless, they remain a useful and practical reference based on precise and comparable data that can complement information obtained through traditional ecological studies of the biological, ecological and functional diversity of the assemblage of reef fish and hermatypic corals of a given location. The Atlantic and Gulf Rapid Reef Assessment (AGRRA) program and the Caribbean Coastal Marine Productivity Program (CARICOMP) use different metrics in order to evaluate the condition of health of the reefs; they not only use total live coral cover, but also consider coral colony size and height, incidence of disease, incidence of bleaching, abundance of bioturbator organisms such as *Diadema*, number of larvae and juveniles of reef fish, survival and growth rates of organisms and taxonomic and functional groups. The CARICOMP assesses the productivity, water quality, species richness of important benthic groups such as sponges, ascidians, as well as the coverage of hard corals, soft corals and several algal groups [19,20,64]. In this sense, evaluations of diversity of the most important groups of organisms of the benthos and reef fish are commonly used, since these provide valuable information regarding the structure and assemblage of the benthic community, as well as its relationship with the assemblage of reef fish, and can thus indicate the condition of the coral reef itself [43,44,47,65,66].

To date, the RHI and 2D-CHI have not been evaluated in relation to the metrics of biological, ecological and functional diversity in order to verify the correct functionality and applicability of these indices for decision-making and the generation of recommendations in reef management. Our analyses showed that, throughout the reefs of the MAR and of the western-central Caribbean Sea, high variation exists in most of the metrics of biological, ecological and functional diversity of fish and hermatypic corals at both site and zone scales (Models 1 and 2). However, only the health grades of the RHI provide relevant information in terms of the biological, ecological and functional diversity of the assemblages of reef fish, where species richness is directly proportional to the health grades assigned by the RHI and higher RHI values represent high species richness and diversity of fish functional groups. This is not applicable to the assemblages of hermatypic corals, however, since neither of the two indices are not related to the metrics of biological, ecological and functional diversity utilized in the present study. Finally, if our interest is to improve our ability to accurately evaluate the health condition of coral reefs, we suggest adoption of both of these health indices (RHI and 2D-CHI) in conjunction with classic community descriptors, as a source of complementary information [32, 42, 44, 66, 67] or well, the diversity indices of fish and corals should be included in the estimation of health indices. It is also necessary to conduct efforts towards systemic analysis (e.g. trophic functioning assessment [68], Network Analysis [69,70] and Loop Analysis [71]) of the reefs supported in this type of strategy. In this manner, it will be possible to achieve a better understanding of the complexity of the bentho-pelagic communities of these coral reef ecosystems, through connection of all of their components that will allow estimation of the health at ecosystem level, as well as that of the most sensitive components of the system, which deserve more detailed attention.

## Supporting Information

S1 Fig Hierarchical sampling design of coral reef zones and sites at Western Caribbean Sea. **Cancun:** Bonanza (Bo), Chitales (Ci), Cuevones (Cu), El Bajito (EB), La Bocana (LB), Manchones (Ma), Punta Nizuc (PN), Radio Pirata (RP); **Banco Chinchorro:** Six sites for Baliza (Ba1, Ba2, Ba3, Ba4, Ba5, Ba6), six sites for Chancay (Ch1, Ch2, Ch3, Ch4, Ch5, Ch6), six sites for Colorados (Co1, Co2, Co3, Co4, Co5, Co6), six sites for Cueva Tiburones (CT1, CT2, CT3, CT4, CT5, CT6), six sites for La Caldera (LC1, LC2, LC3, LC4, LC5, LC6), and six sites for Penelope (Pe1, Pe2, Pe3, Pe4, Pe5, Pe6); **Xcalak:** six sites for Alejandro Reefs (Al1, Al2, Al3, Al4, Al5, Al6), six sites for Hobná (Ho1, Ho2, Ho3, Ho4, Ho5, Ho6), six sites for Bacalar Chico (Bac1, Bac2, Bac3, Bac4, Bac5, Bac6), six sites for Poza Rica (PZ1, PZ2, PZ3, PZ4, PZ5, PZ6), and six sites for Rio Huach (RH1, RH2, RH3, RH4, RH5, RH6); **Punta Manabique:** Piedra de la barracuda (PBA), Bajo del Cabo (BCA), Motaguilla (Mo); **Cayos Cochinos:** three sites for Pelicanos (PL1, PL2, PL3), three sites for Grupera (Gr1, Gr2, Gr3), two sites for Mariposales (Mr1, Mr2), two sites for Salamandinga (Sa1, Sa2), and two sites for Roatan Bank (RB1, RB2); **Media Luna:** six sites for Media Luna (ML1, ML2, ML3, ML4, ML5, ML6) and **Cayos Miskitos:** London Reef (LOR), Nee Reef (NER), Porgee Reef (POR), Lamarka Reef (LAR), Martinez Reef (MAR), Cayo Muerto (MUK). Parentheses indicate the year of sampling for each zone.
(TIF)

S1 File Database of multi-scale analysis. (XLSX)

S1 Table Functional groups of reef fish according to Opitz (1996). (DOCX)

S2 Table RHI health grades according to limit values for each indicator, according to Healthy Reefs for healthy people Initiative (HRI, 2012, 2105). (DOCX)

S3 Table Health grades according to 2D-CHI values. Taken from Kaufman *et al*. (2011).
(DOCX)
