# Correction: Coral Reef Health Indices *versus* the Biological, Ecological and Functional Diversity of Fish and Coral Assemblages in the Caribbean Sea

**DOI:** 10.1371/journal.pone.0167252

**Published:** 2016-11-18

**Authors:** Leopoldo Díaz-Pérez, Fabián Alejandro Rodríguez-Zaragoza, Marco Ortiz, Amílcar Leví Cupul-Magaña, Jose D. Carriquiry, Eduardo Ríos-Jara, Alma Paola Rodríguez-Troncoso, María del Carmen García-Rivas

There are errors in the Author Contributions. The correct contributions are: Conceptualization: LDP FARZ JDC. Data curation: LDP. Formal analysis: LDP FARZ. Funding acquisition: FARZ JDC MCGR. Investigation: LDP FARZ JDC MO ALCM ERJ APRT. Methodology: LDP FARZ JDC. Project administration: FARZ JDC MCGR. Resources: FARZ JDC MCGR. Software: LDP FARZ. Writing – original draft: LDP FARZ. Writing – review & editing: LDP FARZ JDC MO ALCM ERJ APRT.
