# Supplementary figures and images for: Coral Reef Health Indices versus the Biological, Ecological and Functional Diversity of Fish and Coral Assemblages in the Caribbean Sea
Source: PLoS One. 2016 Aug 31;11(8):e0161812. doi: 10.1371/journal.pone.0161812 (PMC5007032; doi:10.1371/journal.pone.0161812)

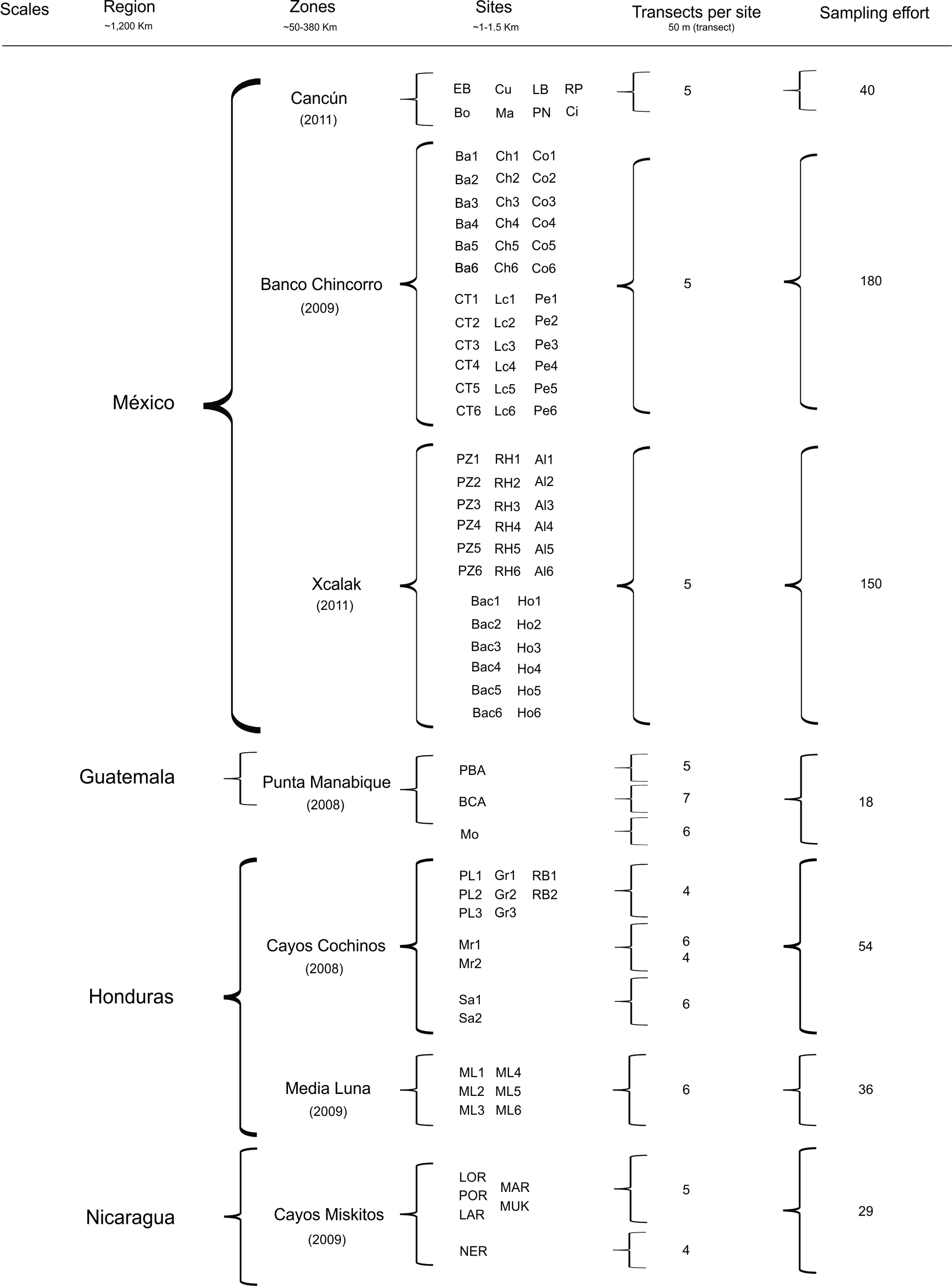

Supplement: S1 Fig — Cancun: Bonanza (Bo), Chitales (Ci), Cuevones (Cu), El Bajito (EB), La Bocana (LB), Manchones (Ma), Punta Nizuc (PN), Radio Pirata (RP); Banco Chinchorro: Six sites for Baliza (Ba1, Ba2, Ba3, Ba4, Ba5, Ba6), six sites for Chancay (Ch1, Ch2, Ch3, Ch4, Ch5, Ch6), six sites for Colorados (Co1, Co2, Co3, Co4, Co5, Co6), six sites for Cueva Tiburones (CT1, CT2, CT3, CT4, CT5, CT6), six sites for La Caldera (LC1, LC2, LC3, LC4, LC5, LC6), and six sites for Penelope (Pe1, Pe2, Pe3, Pe4, Pe5, Pe6); Xcalak: six sites for Alejandro Reefs (Al1, Al2, Al3, Al4, Al5, Al6), six sites for Hobná (Ho1, Ho2, Ho3, Ho4, Ho5, Ho6), six sites for Bacalar Chico (Bac1, Bac2, Bac3, Bac4, Bac5, Bac6), six sites for Poza Rica (PZ1, PZ2, PZ3, PZ4, PZ5, PZ6), and six sites for Rio Huach (RH1, RH2, RH3, RH4, RH5, RH6); Punta Manabique: Piedra de la barracuda (PBA), Bajo del Cabo (BCA), Motaguilla (Mo); Cayos Cochinos: three sites for Pelicanos (PL1, PL2, PL3), three sites for Grupera (Gr1, Gr2, Gr3), two sites for Mariposales (Mr1, Mr2), two sites for Salamandinga (Sa1, Sa2), and two sites for Roatan Bank (RB1, RB2); Media Luna: six sites for Media Luna (ML1, ML2, ML3, ML4, ML5, ML6) and Cayos Miskitos: London Reef (LOR), Nee Reef (NER), Porgee Reef (POR), Lamarka Reef (LAR), Martinez Reef (MAR), Cayo Muerto (MUK). Parentheses indicate the year of sampling for each zone. (TIF) [file pone.0161812.s001.tif]
