# Supplementary material for: Coral Reef Health Indices versus the Biological, Ecological and Functional Diversity of Fish and Coral Assemblages in the Caribbean Sea
Source: PLoS One. 2016 Aug 31;11(8):e0161812. doi: 10.1371/journal.pone.0161812 (PMC5007032; doi:10.1371/journal.pone.0161812)
Supplement: S1 Table — (DOCX) [file pone.0161812.s003.docx]

Table S1. Functional groups of reef fish according to Opitz (1996).

| Group | Fish species | Group | Fish species |
| --- | --- | --- | --- |
| 1 | Large sharks/rays, carnivorous | 15 | Small schooling fish, pelagic |
| 2 | Sharks/Scombrids, carnivorous | 16 | Engraulidae, herbivorous |
| 3 | Large jacks, carnivorous | 17 | Small reef fish, carnivorous 2 |
| 4 | Intermediate jacks, carnivorous | 18 | Large groupers, carnivorous |
| 5 | Small jacks, carnivorous | 19 | Intermediate reef fish, carnivorous 4 |
| 6 | Intermediate reef fish, carnivorous 1 | 20 | Small reef fish, omnivorous 1 |
| 7 | Large intermediate schooling fish, pelagic | 21 | Small reef fish, omnivorous 2 |
| 8 | Intermediate reef fish, carnivorous 2 | 22 | Small reef fish, omnivorous 3 |
| 9 | Hemiramphidae herbivorous | 23 | Large Scaridae, herbivorous |
| 10 | Kyphosidae, herbivorous | 24 | Intermediate Scaridae, herbivorous |
| 11 | Intermediate reef fish, herbivorous | 25 | Small Scaridae, herbivorous |
| 12 | Large reef fish, carnivorous | 26 | Blenniidae, herbivorous |
| 13 | Intermediate reef fish, carnivorous 3 | 27 | Small Gobiidae, carnivorous |
| 14 | Small reef fish, carnivorous 1 |  |  |
