# Supplementary material for: Coral Reef Health Indices versus the Biological, Ecological and Functional Diversity of Fish and Coral Assemblages in the Caribbean Sea
Source: PLoS One. 2016 Aug 31;11(8):e0161812. doi: 10.1371/journal.pone.0161812 (PMC5007032; doi:10.1371/journal.pone.0161812)
Supplement: S2 Table — (DOCX) [file pone.0161812.s004.docx]

S2 Table. RHI health grades according to limit values ​​for each indicator, according to Healthy Reefs for healthy people Initiative (HRI, 2012, 2105)

| RHI Category  (Indicators) | Very Good  (5) | Good  (4) | Fair  (3) | Poor  (2) | Critical  (1) |
| --- | --- | --- | --- | --- | --- |
| Coral cover % | ≥40 | 20 – 39.9 | 10 – 19.9 | 5 – 9.9 | <5 |
| Fleshy algae cover % | 0 – 0.9 | 1 - 5 | 5.1 - 12 | 12.1 - 25 | >25 |
| Herbivorous fish biomass (g 100m^-2^) | ≥3,480 | 2880 – 3,479 | 1920 - 2879 | 961 - 1919 | <960 |
| Commercial fish biomass (g 100m^-2^) | ≥1,680 | 1260 – 1,679 | 840 - 1259 | 421 - 839 | <420 |
