# Supplementary material for: Coral Reef Health Indices versus the Biological, Ecological and Functional Diversity of Fish and Coral Assemblages in the Caribbean Sea
Source: PLoS One. 2016 Aug 31;11(8):e0161812. doi: 10.1371/journal.pone.0161812 (PMC5007032; doi:10.1371/journal.pone.0161812)
Supplement: S3 Table — Taken from Kaufman et al. (2011). (DOCX) [file pone.0161812.s005.docx]

Table S3. Health grades ​​according to 2D-CHI values. Taken from Kaufman *et al.* (2011)

| Value of 2D-CHI | Coral health grade |
| --- | --- |
| 0.81–1.00 | Very healthy |
| 0.61–0.80 | Healthy |
| 0.41–0.60 | Fair |
| 0.21–0.40 | Degraded |
| 0–0.20 | Very degraded |
